# Quantum‐Like Dynamics in Whole‐Brain Models of the Human Connectome

**DOI:** 10.1002/advs.77103

**Published:** 2026-08-18

**Authors:** Gustavo Deco, Yonatan Sanz Perl, Natasha Greenstein, Shamil Chandaria, Gregory Scholes, Morten L. Kringelbach

**Affiliations:** ^1^ Center For Brain and Cognition Computational Neuroscience Group Faculty of Medicine and Life Sciences Universitat Pompeu Fabra Barcelona Spain; ^2^ Institució Catalana de la Recerca i Estudis Avançats (ICREA) Barcelona Spain; ^3^ International Centre for Flourishing Universities of Oxford (UK), Aarhus (Denmark) and Pompeu Fabra (Spain ); ^4^ Department of Engineering Universidad De San Andrés Buenos Aires Argentina; ^5^ Department of Physics Princeton University Princeton New Jersey USA; ^6^ Centre For Eudaimonia and Human Flourishing, Linacre College University of Oxford Oxford UK; ^7^ Department of Psychiatry University of Oxford Oxford UK; ^8^ Department of Chemistry Princeton University Princeton New Jersey USA; ^9^ Center For Music in the Brain Department of Clinical Medicine Aarhus University Aarhus Denmark

## Abstract

Emerging research provides evidence for quantum‐like (QL) probability laws, including interference effects, in non‐quantum physical systems using coupled oscillators. In principle, such systems could produce QL states able to support QL computation in biological tissue. In this perspective, we propose a new avenue of research, namely to investigate if this is true in the human brain. Intriguingly, the special topology of human brain anatomy could promote the rich dynamical repertoire in human cognition. As a proof‐of‐principle, we investigated this by constructing two whole‐brain models with and without QL dynamics, where the QL regime is distinguished by a larger whole‐brain spectral gap. In accordance with our hypothesis, we found that the QL regime provides a significantly better fit to large‐scale human empirical neuroimaging data and exhibits a lower model‐derived energy cost than the non‐QL model. Furthermore, we constructed a whole‐brain model measuring the Växjö Interference term, a measure of functional quantum‐like interference showing a significant correlation with the size of the spectral gap. Overall, these results suggest that quantum‐like whole‐brain models may provide a useful description of large‐scale resting‐state brain dynamics, and we set out what would be required to test whether this description also carries explanatory value for brain function.

## Graphical abstract

Quantum‐like dynamics in the human brain. The level of quantum‐like behavior in a non‐quantum system of coupled oscillators is regulated by the spectral gap of the coupling graph. Whole‐brain modelling using QL fits the empirical data significantly better and has a lower model‐derived energy cost than the non‐QL model.

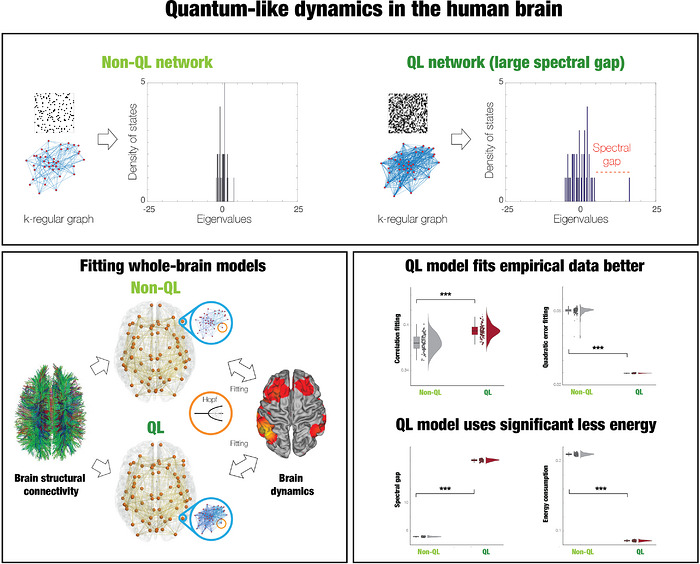

## 1 Introduction

The human brain performs sophisticated computation on a budget of roughly twenty watts [1, 2]. Present‐day artificial intelligence systems accomplish less with orders of magnitude more power. The brain also computes with slow and noisy components, and does so in a distributed manner. How it achieves this combination of flexibility, speed and energy efficiency is an open question with consequences well beyond neuroscience.

In this *Perspective* we propose a new avenue of research. The proposal is that the human brain may exploit quantum‐like (QL) dynamics, meaning interference and superposition of cluster‐synchronized states in networks of classical oscillators, and that these dynamics offer a candidate framework within which such questions can be posed. No microscopic quantum mechanism is invoked. The *quantum* content of the claim is formal and resides solely in the probability laws that certain macroscopic classical oscillator networks are known to satisfy.

Instead, we use the term *quantum‐like* in a precise and deliberately restricted sense. The claim is formal and macroscopic and invokes no microscopic quantum coherence in neural tissue. Following the work of Scholes [3, 4, 5] and the Växjö interpretation of Khrennikov [6], a system is quantum‐like when the probabilities of its measurement sequences cannot be reconstructed within a single classical probability space and instead require a vector description in Hilbert space, so that alternatives compose by interference rather than by additive probability. The operational signatures are therefore the familiar departures from classical probability, namely interference between alternatives together with contextuality and order effects in sequential measurement, and not the mere presence of structured or metastable dynamics. Within our oscillator networks these signatures become available when a spectral gap separates a robust two‐state synchronization mode from the bulk of the spectrum, and two such gapped networks coupled together constitute a quantum‐like bit.

Before we continue, it is important to be clear about what each level of this construction does and does not establish. The spectral gap is a necessary structural condition for the quantum‐like regime and a convenient index of its strength, but it is a generic feature of many classical systems and is not by itself evidence of quantum‐like computation. Hilbert‐space representability shows that the dynamics can be written in the same algebra as a quantum system, which is a statement about form and not about mechanism. The properly quantum‐like content is the non‐classical probability structure that the gapped coupled networks support, and its signature is interference, understood as a violation of the classical law of total probability. As a preliminary proof‐of‐principle, we quantify this signature directly from brain dynamics through the Växjö Interference Connectivity, which we report alongside the improvement in fit and the reduction in modelled energy that the gapped structure confers. We treat this interference measure as a first demonstration rather than as settled evidence, and we separate it throughout from the stronger claim that the brain uses quantum‐like dynamics for cognition, which remains a hypothesis for future work.

**FIGURE 1:**
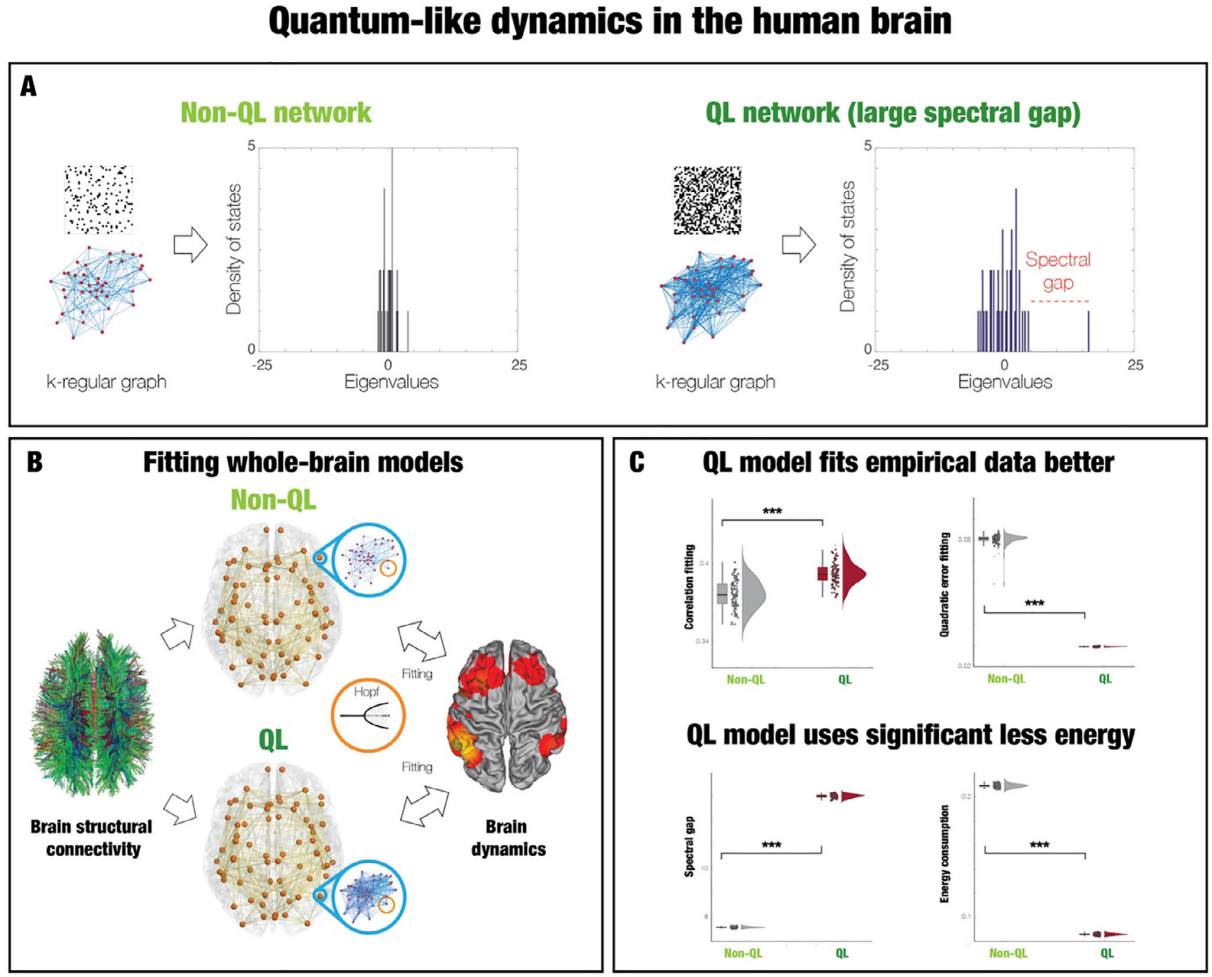
Framework for discovering quantum‐like (QL) dynamics in the human brain. (A) The level of quantum‐like behaviour in a system of coupled oscillators is regulated by the spectral gap of the coupling graph, i.e. the difference between the largest eigenvalues and bulk of the eigenvalues. Specifically in a k‐regular graph this can be achieved by changing the edge density (compare the left and right graphs). (B) These two kinds of systems (QL and non‐QL) can then be used at the regional level in a whole‐brain model and fitted to empirical data of large number of participants. (C) This allows for a direct comparison of the goodness of fit for a number of crucial parameters, showing that the QL model fits the empirical data significantly better and has a lower model‐derived energy cost than the non‐QL model.

Overall, three strands of existing evidence converge on the proposal (Figure 1). The first is from physics. Recent work has shown that a graph and its corresponding connectivity matrix have “spectral gaps”, which are the quantitative guarantee that the emergent synchronized mode is distinct, robust and usable for certain classes of coupled‐oscillator networks to produce emergent probability distributions, robust to noise, that reproduce the statistical signatures of quantum‐like states [3, 4, 5]. Working within the Växjö interpretation of quantum mechanics [6], this line of work establishes that QL states are vector‐like descriptions of measurement sequences and that networks whose largest eigenvalue is well separated from the rest of the spectrum sustain resilient two‐state behavior. Two such networks, coupled, constitute a quantum‐like bit. The result opens the possibility that interference and superposition are not exclusively quantum phenomena but can emerge at macroscopic scales in any medium that supports coupled oscillators, including biological tissue.

The second strand concerns the architecture of the human brain. The human structural connectome possesses spectral gaps, which characterize the algebraic connectivity and associated emergent states, acting as the structural skeleton underlying interference. Adding to this, rare long‐range connections breaking the exponential distance rule are a distinctive feature of mammalian brain anatomy [7, 8, 9, 10]. This architecture scales with brain size. The mouse cortical connectome is ultra‐dense and close to all‐to‐all connected [11], whereas the macaque interareal network is markedly sparser at 66% density, with the long‐range connections carrying the specificity of each area's connectivity profile and being particularly prevalent in the prefrontal cortex [12]. The human cortex is sparser still, with correspondingly more of these long‐range exceptions than in any other species, and it is these weight‐distance relations that make brain architecture unique among known physical systems [7]. Taken together, this allows the brain to shape large‐scale dynamics in ways that amplify the criticality necessary for efficient, fast and turbulent information processing [13].

The third strand is whole‐brain modelling. Classical coupled oscillators such as Stuart‐Landau units fit empirical neuroimaging data [14] and explain the emergence of metastability, the rich dynamical repertoire required for complex cognition [15, 16]. The same analytical framework now makes it possible to quantify the energy of brain computation from neuroimaging data [17].

This *Perspective* proposes to unify these three strands of physics, anatomy and computational modelling, which have developed largely independently. If each cortical region is modelled as a network of coupled oscillators in the QL regime and the regions are in turn coupled by the anatomical connectome [14, 18, 19, 20], the resulting network of networks distributes QL bits across pairs of brain regions. Long‐range connections become the channels along which interference effects propagate. The framework inherits both the analytical machinery of whole‐brain modelling and the expander‐graph resilience that sustains QL states against noise [21]. The axioms that identify QL states, the Hilbert‐space formalism and the linearization of the oscillator dynamics [22] are all given in the *Supplement*.

## 2 Proof of Concept: Quantum‐Like Effects in Empirical Brain Dynamics

**FIGURE 2:**
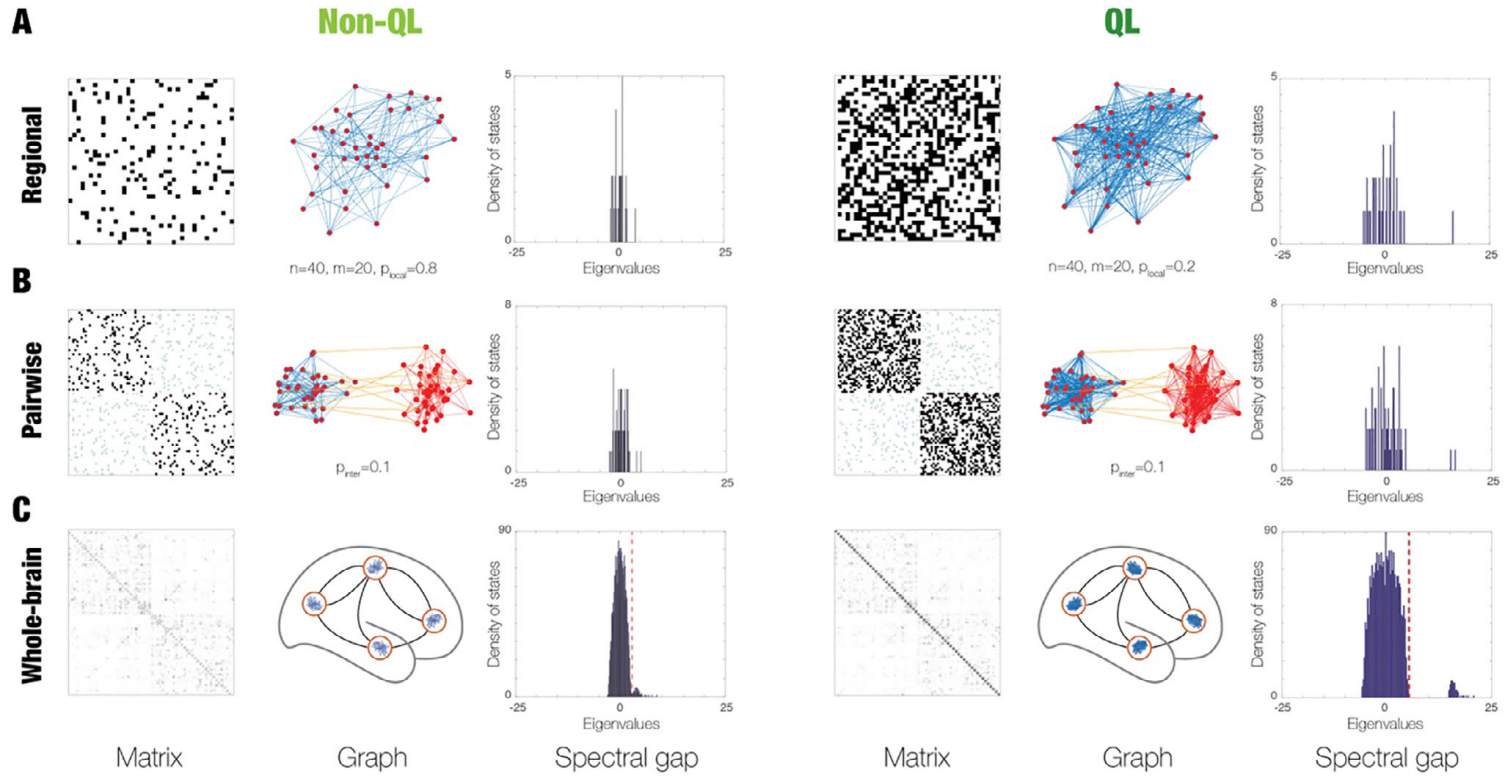
Schematic of the whole‐brain modelling with and without quantum‐like effects. By using different levels of pruning, coupled oscillators in a k‐regular graph can be made to show both non‐QL and QL effects. Here we show the construction of whole‐brain models with non‐QL (left column) and QL (right column) effects at three levels: regional, pairwise and whole‐brain, where for each level we show the connectivity matrix, corresponding graph and resulting spectral gap. (A) For each region (left column) we have a k‐regular graph with high levels of pruning, which does not show QL effects, since it does not show a spectral gap in the eigenvalues of the matrix. In contrast (in the right column) for low levels of pruning, this leads to a spectral gap. (B) A QL bit is typically defined pairwise as the interconnection of two k‐regular graphs. Left column show the results of using a coupling of non‐QL graphs with the corresponding matrix and spectral gap. Comparing this with the results of using QL graphs (in the right column), this gap is significantly larger. C) Defining a network of networks with distributed bits across the whole brain leads to a significantly larger spectral gap when using QL regional graphs as the building blocks for each brain region.

**FIGURE 3:**
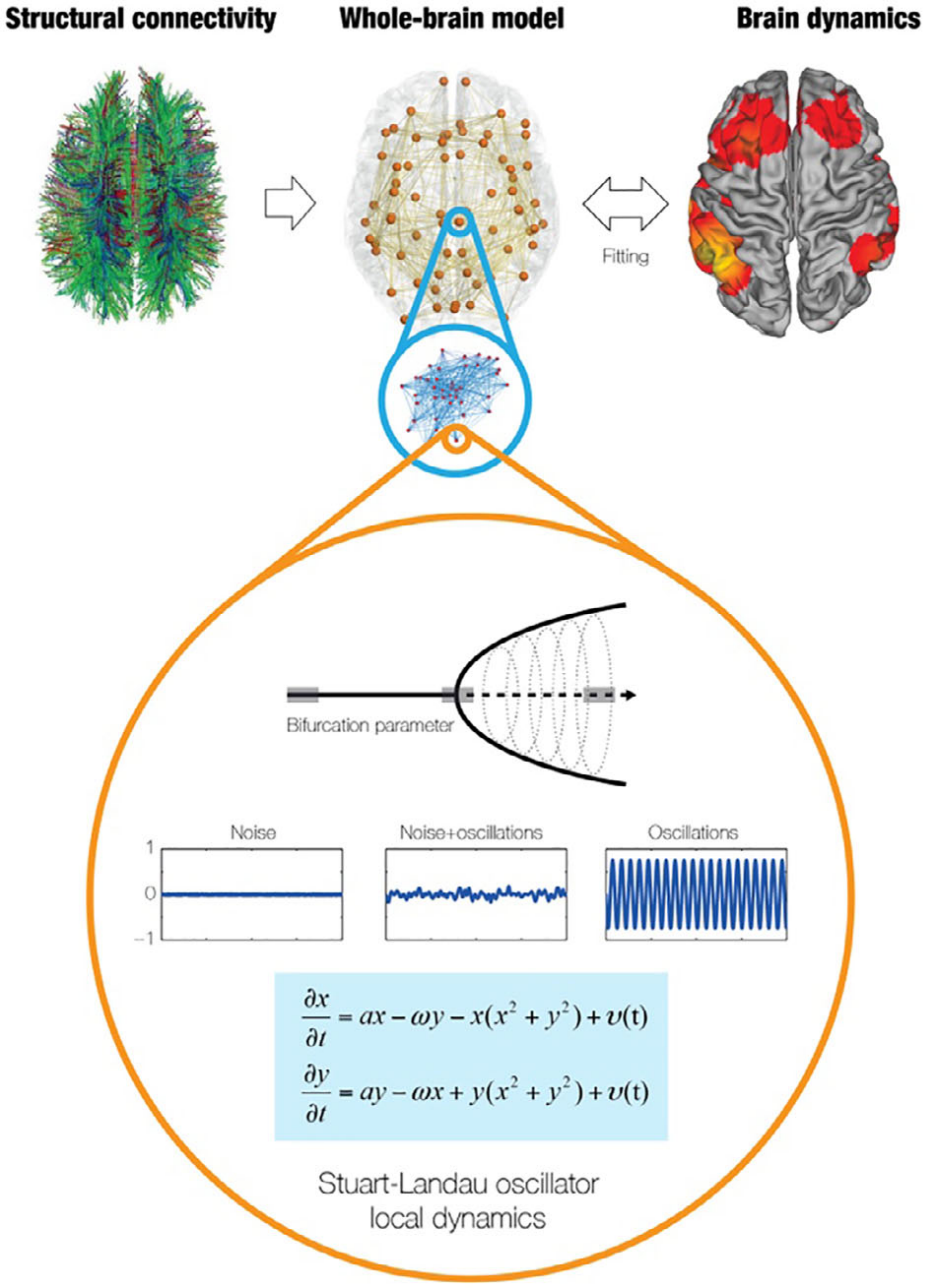
Proof of concept: Whole‐brain model with k‐regular graphs in each brain region. The dynamics of each brain region is governed by a k‐regular network with coupled Stuart‐Landau (SL) oscillators connected with the anatomical structural connectivity and as a result this distributes QL bits across paired brain regions and thus distributed, connected and overlapping across the whole brain.

**FIGURE 4:**
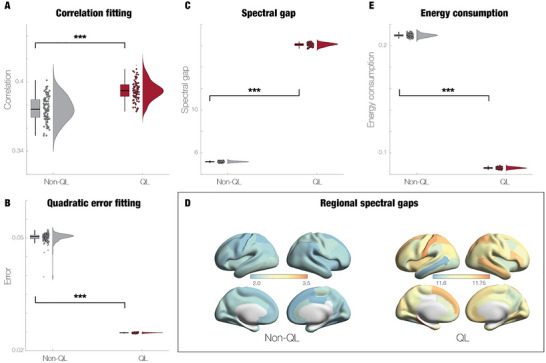
Evidence from quantum‐like whole‐brain modelling of the human brain. (A) Fitting of whole‐brain models with non‐QL and QL local dynamics show significant differences in the level of fitting of large‐scale human neuroimaging resting state data in terms of correlation the simulated and empirical FC matrices (*p* < 0.001, Wilcoxon). (B) The quadratic error fitting is also significantly better for the QL whole‐brain model (*p* < 0.001, Wilcoxon). (C) Significant differences in the underlying spectral gaps shown in the violin plots (*p* < 0.001, Wilcoxon). (D) Renderings of the regional spectral gaps on the surface of the human brain for both non‐QL and QL. E) Significant differences in the underlying energy consumption shown in the violin plots (*p* < 0.001, Wilcoxon).

Briefly, as proof‐of‐principle, and with all technical details in the *Supplement*, we constructed two whole‐brain models, one using the QL regime and the other using non‐QL (Figures 2 and 3). Both were fitted to resting‐state neuroimaging data from over a thousand human participants, and we compared how well each reproduced the empirical functional connectivity (FC) matrices. The QL model fits the empirical data significantly better than the non‐QL model, both in the correlation between simulated and empirical connectivity (Wilcoxon test indicated that the scores for QL (Median = 0.39, IQR = [0.39, 0.40]) were significantly different from Non‐QL (Median = 0.38, IQR = [0.37, 0.38]), Z = −9.25, *p* < 0.001, Cliff's delta = −0.76, Figure 4A) and in the quadratic error (Wilcoxon test indicated that the scores for QL (Median = 0.02, IQR = [0.02, 0.02]) were significantly different from Non‐QL (Median = 0.05, IQR = [0.05, 0.05]), Z = 12.22, *p* < 0.001, Cliff's delta = 1.00, *p* < 0.001, Figure 4B). The difference is driven by the underlying spectral gap, which is significantly larger in the QL regime than in the non‐QL one (Wilcoxon test indicated that the scores for QL (Median = 16.11, IQR = [16.00, 16.21]) were significantly different from Non‐QL (Median = 5.17, IQR = [5.14, 5.21]), Z = −12.22, *p* < 0.001, Cliff's delta = −1.00, Figure 4C).

Projecting the regional spectral gaps onto the cortical surface reveals clear differences between the two regimes and, in the QL case, substantial spatial heterogeneity that hints at variability in the strength of quantum‐like effects across brain regions (Figure 4D).

We also quantified the energy cost of computation in each model using the COCO framework [17], which integrates stochastic thermodynamics with whole‐brain modelling to compute energy consumption, entropy production and information processing analytically from neuroimaging data (see *Supplement*). Within this framework, the QL regime has a substantially lower model‐derived energy cost than the non‐QL regime (Wilcoxon test indicated that the scores for QL (Median = 0.09, IQR = [0.09, 0.09], were significantly different from Non‐QL (Median = 0.21, IQR = [0.21, 0.21]), Z = 12.22, *p* < 0.001, Cliff's delta = 1.00, Figure 4E), reflecting a lower thermodynamic cost of information processing and communication in the Landauer sense within this framework [17].

Adding to this evidence of QL effects in biologically realistic brain dynamics, we also investigated connectome‐constrained whole‐brain models endowed with empirically validated local dynamics. This complementary strategy allowed us to test whether QL effects also emerge naturally at the dynamical regime that best reproduces large‐scale human brain activity. We built a classic Hopf whole‐brain model operating near the operating near the critical bifurcation and constrained by the structural connectome, which has been shown to be a biologically grounded and empirically validated description of large‐scale resting‐state dynamics [18].

**FIGURE 5:**
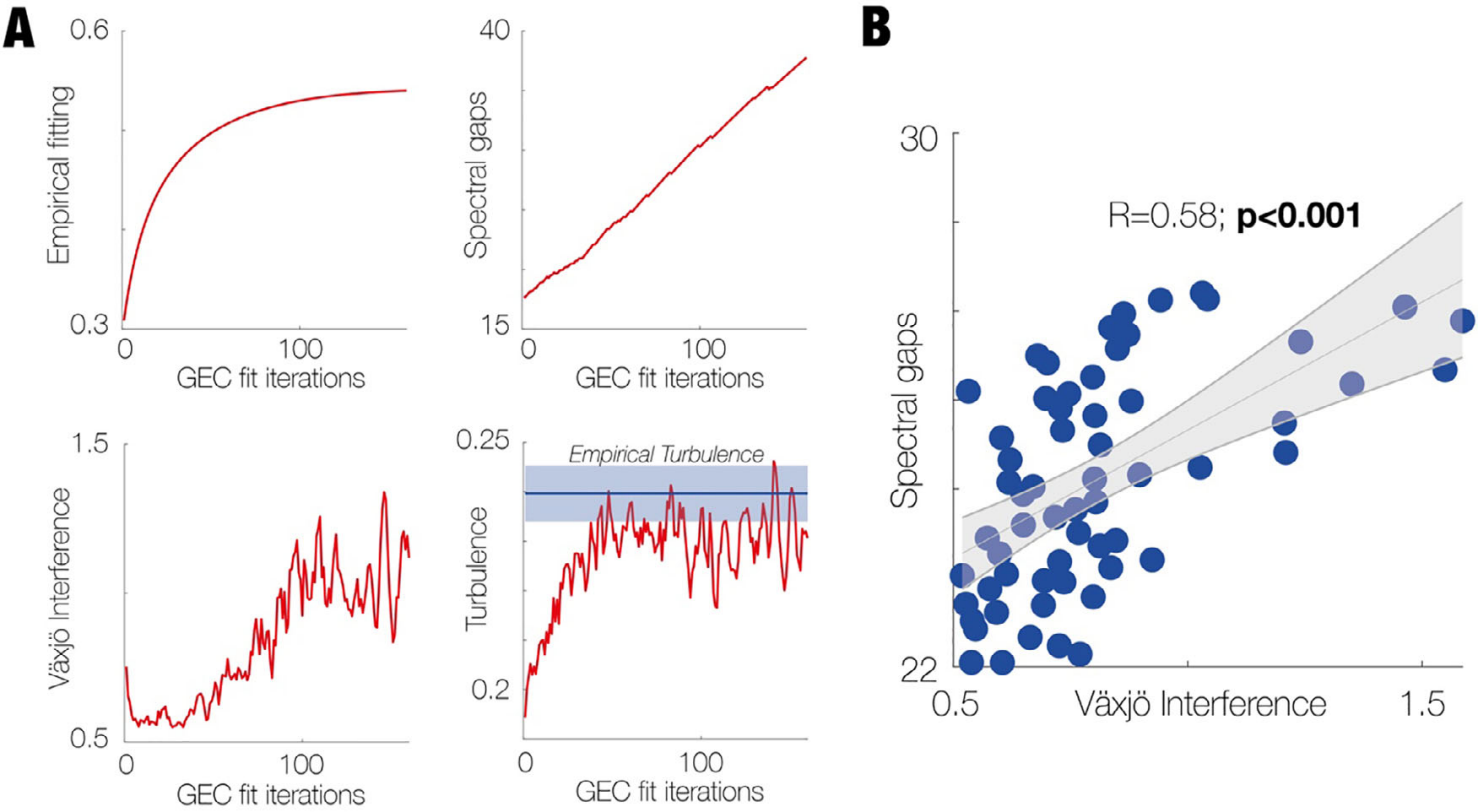
Evidence for functional and structural quantum‐like effects in a whole‐model of brain dynamics. We built a whole‐brain model of the turbulent vortex space, representing instantaneous levels of local synchronization. This allowed us to define a measure of entanglement in brain dynamics as Växjö interference. This quantified interference in brain dynamics as the violation of the law of total probability within the turbulent vortex space [66, 67]. (A) The first panel shows the level of model fitting to empirical large brain dynamics across 1003 participants against the evolution of the iterative improvement of the whole‐brain's generative effective connectivity which determines the level of fitting. The second panel shows that as the model gets better at fitting the empirical data, the spectral gaps increase. The third panel, show how the level of Växjö interferences increase. The fourth panel shows level of turbulence in the model, which reaches the empirical level of turbulence. (B) Importantly, the model demonstrates a high correlation (R = 0.58, *p* < 0.001) between the functional QL (Växjö interferences) and the structural QL (spectral gaps).

We used this model to quantify the quantum‐like interference directly from the model‐generated brain dynamics. Specifically, we employed the Växjö interference formalism, which characterizes departures from the classical law of total probability and provides a principled measure of contextual probabilistic interactions. The analysis was performed in turbulent vortex space, where instantaneous patterns of local synchronization define the dynamical states of the system. Within this framework, entanglement‐like effects are quantified as interference terms arising from violations of classical probabilistic composition rules. The results reveal a striking convergence between empirical model fitting, spectral organization, and QL dynamical effects (Figure 5A). As the model parameters approached the regime that optimally reproduced empirical resting‐state fMRI data from the 1,003 participants, the spectral gap increased systematically. Critically, the magnitude of Växjö interference increased in parallel, indicating that QL dynamical effects emerge naturally as the model approaches the empirically observed operating point. Thus, the same dynamical regime that best explains large‐scale human brain activity is also characterized by stronger interference effects and enhanced QL spectral organization. Consistent with this interpretation, the structural QL signature, quantified by the spectral gap, was strongly associated with the dynamical QL signature, quantified by Växjö interference (*R*  =  0.58, *p* < 0.001) (Figure 5B). This association is expected rather than coincidental. The spectral gap and the Växjö interference are not independent quantities but two readings of the same self‐adjoint connectome operator under the functional calculus, with the gap acting as their shared control parameter [23]. What the present analysis adds is that the relationship holds in whole‐brain models constrained by empirical anatomy and fitted to human neuroimaging data, rather than only in the k‐regular construction where the spectral gap is set by hand. We note that the correlation does not by itself distinguish quantum‐like organization from the size of the spectral gap alone, a point we return to below.

**FIGURE 6:**
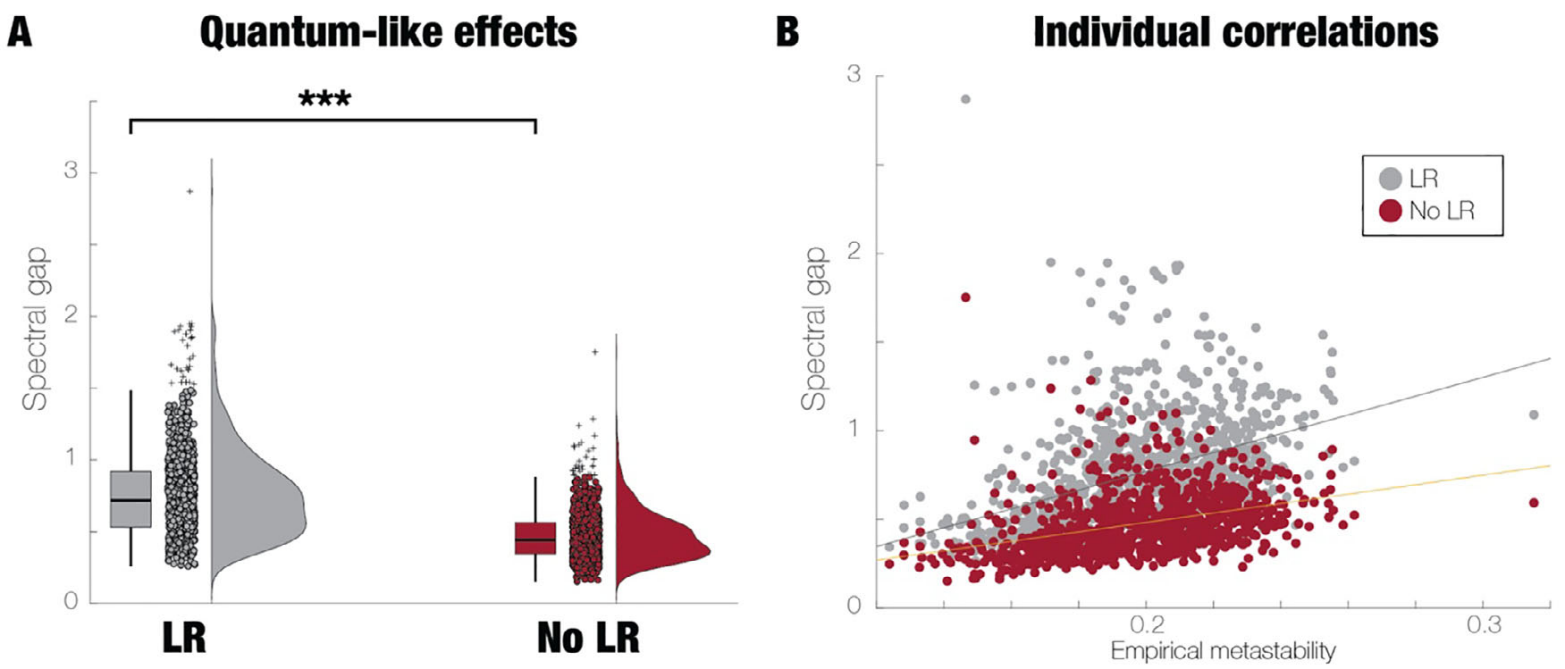
Evidence for the importance of long‐range connections amplifying quantum‐like (QL) dynamics in whole‐brain models of the human brain. (A) There is a significant decrease in spectral gaps when the long‐range exceptions (>80 mm) are removed from the underlying structural anatomical connectivity, showing that long‐range exceptions amplify the quantum‐like effects in the model (*p* < 0.001, Wilcoxon). (B) As seen from the scatter plot, there is a stronger correlation between metastability and the QL effects (measured by spectral gap) between the individualised QL whole‐brain models with (R = 0.42, *p* < 0.001) and without long‐range (R = 0.37, *p* < 0.001) exceptions.

Importantly, we were also able to investigate the contribution of rare long‐range anatomical connections breaking the exponential distance rule, which are a distinctive feature of mammalian brain anatomy [7, 10]. Kennedy and colleagues established this rule by retrograde tract tracing in nonhuman primates, showing that local connectivity is largely explained by geodesic distance across the cortical surface [8, 9]. To test whether these rare long‐range connections matter for QL dynamics, we constructed individualized whole‐brain QL models for each of the 1003 participants using generative effective connectivity, and compared versions with and without long‐range connections. Removing the long‐range connections sharply reduces the spectral gap (Figure 6A), showing that long‐range connectivity amplifies QL dynamics. That the specific placement of these connections matters, and not merely their number or their length distribution, has been shown directly. Comparing four representations of the underlying anatomy, namely cortical geometry, a binary and a continuous exponential distance rule, and the exponential distance rule with rare long‐range exceptions, the last outperforms all others in reconstructing empirical functional connectivity, and a null model in which the long‐range connections are shuffled while the exponential backbone and their number are held fixed cannot reconstruct long‐range functional connectivity to the same extent [10]. Removing these connections from whole‐brain models likewise degrades information processing [7]. We also examined the relationship between QL effects, indexed by the spectral gap and metastability, a measure of dynamical repertoire (see Supplement). The correlation between metastability and spectral gap is significantly stronger in models with long‐range connections (R = 0.42, *p* < 0.001) than in those without (R = 0.37, *p* < 0.001) as shown in Figure 6B, suggesting that metastability, expressed as variability in cluster synchronization, is dynamically linked to the underlying QL spectral structure and is enhanced by long‐range anatomical connectivity.

## 3 Discussion

Here we have proposed that the human brain may exploit quantum‐like (QL) dynamics and have offered an initial test using whole‐brain modelling with and without QL effects. The whole‐brain models using classical coupled oscillators provide a significantly better fit to empirical brain activity when incorporating QL dynamics than without them, as indexed by differences in spectral gaps. The QL whole‐brain models are also significantly more energy efficient than their non‐QL counterparts. These findings are consistent with our hypothesis but should be read as proof‐of‐principle rather than conclusive evidence of QL computation in the brain.

What makes the proposal a candidate specifically for the mammalian brain is its distinctive cortical architecture. Rare long‐range connections that break the exponential distance rule [8, 9] amplify QL effects in the model, and removing them sharply reduces the spectral gap [7, 10]. In our simulations, the strength of QL effects tracks metastability, the richness of the dynamical repertoire that supports complex cognition [14, 16, 24] and that is a sensitive marker of neurological and psychiatric disease [25]. If borne out, QL dynamics would sit at the structural pivot where the distinctive wiring of the human cortex meets the functional signatures of cognitive flexibility and clinical state.

The proposal sits alongside but differs from quantum cognition [26, 27], which imports quantum probability as a formal device for behavioral paradoxes without committing to a physical substrate. Quantum mechanics has transformed our understanding of the microscopic world, particularly through concepts such as nonlocality and entanglement [28, 29, 30]. Here the substrate is concrete, namely coupled oscillators bound by the connectome, and the formalism is inherited from Scholes’ work showing that classical networks can sustain interference and superposition at macroscopic scales [3]. This also inverts a standing concern of quantum information science, where decoherence makes quantum hardware fragile [31] and limits the scalability of devices that rely on interference to compute [32, 33, 34, 35]. QL systems arise in a classical macroscopic regime that appears robust to noise and disorder, and may therefore suggest design principles for energy‐efficient classical computation that does not depend on refrigeration or environmental isolation.

Our framework is also close in spirit to a body of work by Khrennikov and colleagues that derives quantum‐like structure directly from the activity of oscillatory neuronal networks [36] and that represents the joint activity of such networks as quantum‐like states in order to model what they call mental entanglement [37]. The two approaches share the conviction that classical oscillatory dynamics can carry genuinely nonclassical probability structure, and both read that structure through the Växjö interpretation as contextual rather than microscopically quantum. They differ in where they locate the source of the effect. Khrennikov and colleagues build the quantum‐like representation from probabilistic contextuality and from the algebra of observables defined on the network, so that mental entanglement appears as a property of the joint probability structure. We instead derive the quantum‐like regime from the spectral graph theory of the coupling matrix, so that interference and the two‐state structure of a quantum‐like bit follow from the separation between the leading synchronization mode and the bulk of the spectrum. The accounts are therefore complementary. The contextual framework specifies the measurement‐level signatures that a quantum‐like system should display, while the spectral‐gap framework specifies the structural conditions under which a network of coupled oscillators can sustain them. A natural next step is to ask whether the cluster‐synchronization states we describe satisfy the contextual probability laws that Khrennikov and colleagues take as definitive, which would bring the structural and the probabilistic criteria together within a single model.

### 3.1 Important Caveats

The present framework is exact as mathematics, yet at this point still approximate as a description of human brain dynamics. First, we note that a better fit of a whole‐brain model is not a mechanism. Our results show that a whole‐brain model in the quantum‐like regime reproduces resting‐state functional connectivity more accurately, and at lower modelled energy, than a matched model in the non‐quantum‐like regime. The spectral gap distinguishes the two regimes and is a necessary structural condition for quantum‐like dynamics and a useful index of their strength, but it is also a generic property of many classical networks and does not by itself establish that the brain performs quantum‐like computation. A denser or stiffer graph can improve functional‐connectivity agreement for reasons unrelated to interference, and the two regimes here differ in local edge density as a direct consequence of the pruning that defines them. This fact led us to construct a conventional whole‐brain model, in which we found that an established measure of quantum‐like interference, the Växjö interference, correlates with the spectral gaps.

Second, the quantum‐like structure reported here is measured with fMRI and as such a property of large‐scale hemodynamic dynamics. This is a deliberate choice rather than an oversight, since the model is intended as a description of the BOLD signal and not of fast neuronal activity, and the relationship between the BOLD signal and the underlying neural dynamics is well established in the neuroimaging literature [38]. Yet, extending it to fast neuronal activity through an explicit forward model of neurovascular coupling is a natural next step. In the present model, the oscillators are fitted directly in the hemodynamic band, and their real part is read as the BOLD signal, which is the standard practice of Hopf whole‐brain modelling but does not itself constitute a mechanism linking neuronal to hemodynamic timescales. An alternative and complementary approach would model faster oscillators in the neuronal range and pass their activity through a hemodynamic response function, ideally one estimated per region and per participant, and comparing the two schemes is a worthwhile direction for future work. We therefore do not claim that the Stuart‐Landau dynamics fitted here are equivalent to neuronal activity, and the mathematical correspondence between the Hopf normal form and the mean field of quadratic integrate‐and‐fire neurons should be read as a statement about form rather than about the timescale at which the present model operates.

Third, the reduction in energy in the quantum‐like regime is a quantity derived within the stochastic‐thermodynamic framework applied to the whole‐brain model, and it measures the thermodynamic cost of information processing and communication in the Landauer sense rather than metabolic expenditure. We therefore do not equate it with glucose or oxygen consumption, and any mapping onto metabolic measures such as FDG‐PET or calibrated oxygen‐consumption data would require further assumptions and remains a separate question for future work.

Fourth, the relationship reported here between the spectral gap and metastability is correlational. The interference we measure through the Växjö Interference Connectivity is a more direct signature, but we present it as a preliminary proof‐of‐principle, and a null model matched for the spectral gap is needed to establish that the interference reflects specifically quantum‐like organization rather than the size of the gap alone. The causal tests that would connect these dynamical signatures to cognition, for instance pharmacological or stimulation‐based manipulation of long‐range connectivity, remain to be done.

Taken together, what we take to be demonstrated is the modelling claim that a quantum‐like whole‐brain model fits resting‐state data better and at lower modelled energy than a matched nonquantum‐like model. What we infer, more tentatively, is that this is consistent with a role for quantum‐like dynamics in the efficiency and flexibility of large‐scale brain activity. What remains a hypothesis for future work is the biological claim itself, that the brain uses quantum‐like information processing. We have kept these three registers distinct throughout the revised manuscript.

### 3.2 Future Questions

Several questions must be addressed before the proposal can move beyond proof‐of‐principle. The correlational link between QL spectral structure and metastability needs causal tests. For instance, do pharmacological or stimulation‐based manipulations that alter long‐range connectivity change both QL indices and behavior? How the model‐derived Landauer cost of information processing and communication relates to the brain's metabolic budget is a further open question, since the two are related but distinct quantities. Do the boundary conditions of the framework need mapping by identifying which oscillatory regimes in the brain support QL dynamics and which do not? Also, rather than modelling each brain region as QL or non‐QL k‐regular graphs, which allows us to directly test the causal consequences, what would be the consequences of constructing a whole‐brain model in turbulent vortex space (containing the instantaneous levels of local synchronisation) and measuring entanglement in brain dynamics using the Växjö Interference framework? These are tractable and exciting questions within the existing whole‐brain modelling framework, and we offer this *Perspective* as an invitation to pursue them.

## 4 Supplement

### 4.1 Empirical Methodology: Human Connectome Project: Acquisition and Pre‐Processing

#### 4.1.1 Ethics

The Human Connectome project is based on ethics obtained by the Washington University–University of Minnesota (WU‐Minn HCP) Consortium, which obtained full informed consent from all participants. Research procedures and ethical guidelines were followed in accordance with Washington University institutional review board approval (Mapping the Human Connectome: Structure, Function, and Heritability; IRB # 201204036).

#### 4.1.2 Participants

We chose a sample of 1003 participants, all of whom have resting state data. These participants were selected from the March 2017 public data release from the Human Connectome Project (HCP).

#### 4.1.3 3T Structural Data

The HCP structural data were acquired using a customized 3 Tesla Siemens Connectom Skyra scanner with a standard Siemens 32‐channel RF‐receive head coil. For each participant, at least one 3D T1w MPRAGE image and one 3D T2w SPACE image were collected at 0.7 mm isotropic resolution.

#### 4.1.4 Neuroimaging Acquisition for fMRI HCP

All the 1003 HCP participants were scanned on a 3‐T connectome‐Skyra scanner (Siemens). For the resting state data, we used one fMRI acquisition of approximately 15 min acquired on the first day, where participants had eyes open with relaxed fixation on a projected bright cross‐hair on a dark background.

We used the standard minimal pre‐processing of the HCP resting state (as described in full detail on the HCP website). This uses the standard HCP pipeline with standardized methods including FSL (FMRIB Software Library), FreeSurfer, and the Connectome Workbench software [39, 40]. This pre‐processing includes correction for spatial and gradient distortions and head motion, intensity normalization and bias field removal, registration to the T1‐weighted structural image, transformation to the 2 mm Montreal Neurological Institute (MNI) space, and using the FIX artefact removal procedure [40, 41]. Head motion parameters were regressed out, and structured artefacts were removed by Independent Component Analysis followed by FMRIB's ICA‐based X‐noiseifier [42, 43].

The final pre‐processed timeseries are in HCP CIFTI greyordinates standard space and available in the same standard surface‐based CIFTI file for each participant for resting state.

From these pre‐processed timeseries, we extracted the average timeseries in DK62 parcellation with 62 cortical regions (31 regions per hemisphere) [44]. Using a custom‐made Matlab script using the ft_read_cifti function from the Fieldtrip toolbox [45] to extract and average in each region. We filtered these average BOLD timeseries using a second‐order Butterworth filter in the range of 0.008–0.08 Hz.

#### 4.1.5 Human Structural Connectome

In order to reconstruct a high‐quality average structural connectivity (SC) matrix for constructing the whole‐brain model (using the DK62 parcellation), we obtained multi‐shell diffusion‐weighted imaging data from 32 participants from the HCP database (scanned for approximately 89 min). The acquisition parameters are described in detail on the HCP website [46]. The connectivity was estimated using the method described by Horn and colleagues [47].

In summary, the data were processed using a generalized q‐sampling imaging algorithm implemented in DSI Studio (http://dsi‐studio.labsolver.org). A white‐matter mask was produced from segmentation of the T2‐weighted anatomical images, co‐registered the images to the b0 image of the diffusion data using SPM12. For each of the 32 HCP participants, 200 000 fibers were sampled within the white‐matter mask. Fibers were transformed into MNI space using Lead‐DBS [48]. The methods used algorithms for false‐positive fibers shown to be optimal in recent open challenges [49, 50]. Accordingly, the risk of false‐positive tractography was reduced by using the tracking method achieving the highest (92%) valid connection score among 96 methods submitted from 20 different research groups in a recent open competition [49].

### 4.2 Mathematical Framework

#### 4.2.1 Hilbert Space

Briefly, a Hilbert space H is a complete vector space over a field Z. Here we use a real field with the inner product 〈*x*, *y*〉, thereby generalising the Cartesian dot product. So, for any vectors *x*, *y*, *z* in H and with scalars α = ∈*Z*, the following equalities are satisfied:

(1) $$\langle x+y,z\rangle =\langle x,z\rangle +\langle y,z\rangle$$

(2) $$\langle \alpha x,y\rangle =a\langle x,y\rangle$$

(3) $$\langle x,y\rangle =\bar{\langle x,y\rangle }\;$$

(4) $$\langle x,x\rangle =a\geq 0$$

where overbar signifies the complex conjugate. The norm is defined in terms of the inner product: 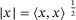.

#### 4.2.2 Whole‐Brain Dynamics

### 4.3 Mathematically, the Whole‐Brain Model Uses Dynamics as Defined by

(5) $$\frac{dz_{j}}{dt}=\left(a_{j}+i\omega_{j}\right)\;z_{j}-{\left|z_{j}\right|}^{2}z_{j}+G\sum_{k=1}^{M}{\hat{C}}_{jk}\left(z_{k}-z_{j}\right)+\eta_{j}$$

where for the oscillator in region *j*, the complex variable *z_j_* denotes the state (*z_j_* = *x_j_*  + i*y_j_*), η_*j*_ is additive uncorrelated Gaussian noise with variance σ^2^ (for all *j*), ω_*j*_ is the intrinsic node frequency, and *a_j_* is the node's bifurcation parameter. Within this model, the intrinsic frequency ω_*j*_ of each node is in the 0.008–0.08 Hz band. The intrinsic frequencies were estimated from the empirical data, as given by the averaged peak frequency of the narrowband blood‐oxygen‐level‐dependent (BOLD) signals of each brain region. For *a_j_* > 0, the local dynamics settle into a stable limit cycle, producing self‐sustained oscillations with frequency ω_*j*_/(2π). For *a_j_* < 0, the local dynamics present a stable spiral point, producing damped or noisy oscillations in the absence or presence of noise, respectively. The fMRI signals were modelled by the real part of the state variables, i.e., *x_j_* =  Real(*z_j_*). The fitting of the whole‐brain model is accomplished by finding the optimal fit of the global coupling parameter (global conductivity), *G*, by minimising the elementwise quadratic error between the functional connectivity matrices (Pearson correlations between the fMRI time series in each brain region) of the model (*FC^model^*) and empirical (*FC^emp^*) data.

This large‐scale optimization is made possible by the linearization of the SL oscillator [22]. Proximity of the SL oscillator to criticality has been shown to provide the best working point for fitting whole‐brain neuroimaging dynamics. This happens at the brink of the bifurcation, i.e., with *a_j_* slightly negative but very near to zero (usually *a_j_* =   − 0.02) [14]. Briefly, we can estimate the functional correlations of the whole‐brain network using a linear noise approximation (LNA). Hence, the dynamical system of *N* nodes (Equation 5) can be re‐written in vector form as:

(6) $$\frac{dz}{dt}=\left(a-S+i\omega \right)⊙z-\left(z⊙\bar{z}\right)z+\hat{C}x+\eta$$

where $z={\left[z_{1},\text{…},z_{M}\right]}^{T}$, $a={\left[a_{1},\text{…},a_{M}\right]}^{T}$, $\omega ={\left[\omega_{1},\text{…},\omega_{M}\right]}^{T}$, $\eta ={\left[\eta_{1},\text{…},\eta_{M}\right]}^{T}$ and $S={\left[S_{1},\text{…},S_{M}\right]}^{T}$ is a vector containing the strength of each node, i.e., $S_{i}=\sum_{j}{\hat{C}}_{ij}$. The superscript []^*T*^represents the transpose, $\bar{z}$ is the complex conjugate of *z* and ⊙ is the Hadamard element‐wise product. As such, the equation describes the linear fluctuations around the fixed point *z*  =  0, which is the solution of $\frac{dz}{dt}=0$. Discarding the higher‐order terms $(z⊙\bar{z})z$ and separating the real and imaginary parts of the state variables, the evolution of the linear fluctuations follows a Langevin stochastic linear equation:

(7) $$\frac{d}{dt}\delta u=J\delta u+\eta$$

where the 2*M*‐dimensional vector $\delta u={\left[\delta x,\delta y\right]}^{T}={\left[\delta x_{1},\text{…},\delta x_{M},\delta y_{1},\text{…},\delta y_{M}\right]}^{T}\;$ contains the fluctuations of real and imaginary state variables. The 2*M* × 2*M* matrix *J* is the Jacobian of the system evaluated at the fixed point, which can be written as a block matrix

(8) $$J=\left[\begin{array}{cc} J_{xx} & J_{xy}\\ J_{yx} & J_{yy} \end{array}\right]\;$$

where *J_xx_*, *J_xy_*, *J_yx_*, *J_yy_* are *M* × *M* matrices given as *J_xx_* = *J_yy_*  =  diag(*a* − *S*) + *C* and *J_xy_* = −*J_yx_*  =  diag(ω), where diag(*v*) is the diagonal matrix whose diagonal is the vector *v*. Please note that this linearisation is only valid if *z*  =  0 is a stable solution of the system, that is if all eigenvalues of *J* have negative real parts. To compute the covariance matrix *K*  = 〈δ*u*δ*u^T^* 〉, one can begin by writing Equation (6) as *d*δ*u*  =  *J*δ*udt* + *dW*, where *dW* is an 2*M*‐dimensional Wiener process with covariance 〈*dWdW^T^* 〉 =  *Qdt*, where *Q* is the noise covariance matrix, which is diagonal if the noise is uncorrelated. Using Itô’s stochastic calculus, we get *d* (δ*u*δ*u^T^*) =  *d*(δ*u*)δ*u^T^* + δ*ud*(δ*u^T^*) + *d*(δ*u*)*d*(δ*u^T^*). Noting that 〈δ*udW^T^* 〉 =  0, taking the expectations and keeping terms to first order in the differential *dt*, we obtain:

(9) $$\frac{dK}{dt}=J\;K+K\;J^{T}+Q$$

Hence, the stationary covariances (for which $\frac{dK}{dt}=0$) can be obtained by solving the following analytic equation:

(10) $$J\;K+K\;J^{T}+Q=0$$

Equation (10) is a Lyapunov equation that can be solved using the eigen‐decomposition of the Jacobian matrix [51]. We obtained the simulated functional connectivity matrix *FC^model^* by reducing (through averaging across the *n* in each brain region) the functional connectivity obtained from the first *M* rows and columns of the covariance *K*, which corresponds to the real part of the dynamics, and thus the BOLD fMRI signal.

#### 4.3.1 Optimizing the Generative Effective Connectivity (GEC)

For defining the GEC of a whole‐brain model, we first fit the model to the empirical neuroimaging data for each participant. For this, we used a noise η_*j*_ with variance σ^2^ =  0.02 homogeneous for all regions in a given parcellation. In order to create the generative effective connectivity (GEC), we used a pseudo‐gradient procedure to optimize the initial coupling connectivity matrix *C*, derived from the anatomical structural connectivity, which was then iteratively used to create the GEC.

Specifically, we iteratively compared the output of the model with the empirical measures of the functional correlation matrix (*FC^empirical^*), i.e., the normalized covariance matrix of the functional neuroimaging data. Furthermore, we also compared the output of the model with the normalized τ time‐shifted covariances (*FS^empirical^* (τ)). These normalised time‐shifted covariance matrices are generated by taking the shifted covariance matrix *KS^empirical^* (τ) and dividing each pair (*i*, *j*) by $\sqrt{KS_{ii}^{empirical}\left(0\right)KS_{jj}^{empirical}\left(0\right)}$.

Note that these normalized time‐shifted covariances break the symmetry of the couplings and thus improve the level of fitting [52]. Using a heuristic pseudo‐gradient algorithm, we proceeded to update the *C* until the fit is fully optimised. More specifically, the updating uses the following form:

(11) $$C_{ij}=C_{ij}+\alpha \left(FC_{ij}^{empirical}-FC_{ij}^{model}\right)+\varsigma \left(FS_{ij}^{empirical}\left(\tau \right)-FS_{ij}^{model}\left(\tau \right)\right)$$

where $FS_{ij}^{model}\left(\tau \right)$ is defined similar to $FS_{ij}^{empirical}\left(\tau \right)$. In other words, the updating is given by the first *N* rows and columns of the simulated τ time‐shifted covariances *KS^model^* (τ) normalised by dividing each pair (*i*, *j*) by $\sqrt{KS_{ii}^{model}\left(0\right)KS_{jj}^{model}\left(0\right)}$, where *KS^model^* (τ) is the shifted generative covariance matrix computed as follows:

(12) $$KS^{model}\;\left(\tau \right)=\exp\left(\tau J\right)\;K\;$$

Please note that *KS^model^* (0) =  *K*. The model was then run repeatedly with the updated *C* until the fit converges towards a stable value. As stated above, we initialised *C* using the average anatomical connectivity and only update known existing connections from this matrix (in either hemisphere). There is one exception, however, to this rule, which is that the algorithm also updates homologue connections between the same regions in either hemisphere, given that tractography is known to be less accurate when accounting for this connectivity.

For the Stuart‐Landau model, we used α  =  ς  =  0.0004 and continued until the algorithm converges. For each iteration, we compute the model results as the average over as many simulations as there are participants. In the human case, we applied this procedure to fit a whole‐brain model to resting state data for each individual, which generated 1003 individual GECs, which were subsequently used in the framework described as follows.

#### 4.3.2 Analytical Derivation of Entropy Production, Energy Dissipation and Information Processing

Nonequilibrium systems are central to many scientific fields, but fundamental challenges remain, especially in defining microscopic entropy production—a key measure of irreversibility. In thermodynamic terms, the change of entropy Δ*S* for a reversible process is given by:

(13) $$\Delta S=\int \frac{dQ}{T}\;$$

where *dQ* is the heat exchanged and *T* is temperature. For irreversible processes,

(14) $$\Delta S\geq \int \frac{dQ}{T}$$

and the difference defines entropy production *H_P_*, i.e.:

(15) $$\Delta S=H_{P}+\int \frac{dQ}{T}$$

The rates of change derived with respect to time (with the dot on the top of a function signifying the time derivative) are given by:

(16) $$\dot{S}\;\left(t\right)=\overset{.}{\underset{P}{H}}\;\left(t\right)-\dot{\phi }\left(t\right)$$

In this equation $\overset{.}{\underset{P}{H}}\left(t\right)$ is the entropy production rate of the system, which (according to the second law of thermodynamics) is always non‐negative, and $\dot{\phi }\left(t\right)$ is the entropy flux rate from the system to the environment. For systems in a non‐equilibrium steady‐state, $\dot{S}\;\left(t\right)=0$ which implies $\overset{.}{\underset{P}{H}}\;\left(t\right)=\dot{\phi }\;\left(t\right)\geq 0$. Please note that it is only for thermodynamic equilibrium that the equality in this equation is expected to hold.

In the brain, $\dot{S}\left(t\right)$, the rate of change of entropy, can be interpreted as “computation”, that is, the flexible mapping of input with output through internal transformations and consequently as a measure of information processing. On the other hand, the $\dot{\phi }\left(t\right)$ is the entropy flux can be interpreted as the energy cost of performing a particular computation, and $\overset{.}{\underset{P}{H}}\left(t\right)$ is the level of non‐equilibrium. We stress that these are interpretations of thermodynamic quantities within the stochastic‐thermodynamic framework, and not established measures of computation or of metabolic expenditure. These fundamental thermodynamic quantities can be calculated analytically when the system is a linear Langevin system, which is exactly the case for our linearised Stuart–Landau model.

Let us by consider the following Langevin system using a simplifying notation of δ*u* **=** *x*:

(17) $$\frac{dx\left(t\right)}{dt}=Jx\left(t\right)+\xi \left(t\right)$$

The ξ(*t*) are 2N independent standard Wiener processes with covariance:

(18)

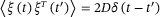




Therefore, *D* is a 2*M* × 2*M* diagonal matrix (but now generalised to the possibility that the diagonal elements could be different). The angular brackets indicate the mathematical expectation over time, and the δ(*t*) is the Dirac's delta function.

Given this, it is straightforward to derive the temporal evolution of the covariance matrix, θ  = 〈*x*(*t*)*x^T^* (*t*′)〉  − 〈*x*(*t*)〉〈*x*(*t*′)〉, which is given by:

(19) $$\frac{d\theta \left(t\right)}{dt}=\theta \left(t\right)J+J\left(t\right)\theta^{T}\left(t\right)+2D$$

The probability distribution *P*(*x*, *t*) associated with the Langevin equation is described by the following Fokker–Planck Equation:

(20) $$\frac{\partial P\left(x,t\right)}{\partial t}=\nabla .\left[-Jx\left(t\right)P\left(x,t\right)+D\nabla P\left(x,t\right)\right]$$

where ∇ denotes the spatial derivative with respect to *x*. The above Fokker–Planck equation can be expressed as the following continuity equation:

(21) $$\frac{\partial P\left(x,t\right)}{\partial t}+\nabla F\;\left(x,t\right)=0$$

where the probability current is given by:

(22) $$F\;\left(x,t\right)=-D\nabla P\left(x,t\right)+Jx\left(t\right)P\left(x,t\right)$$

The stationary solution can be derived analytically and is given by the following multivariate Gaussian distribution:

(23) $$P\;\left(x\right)=\frac{1}{\sqrt{{\left(2\pi \right)}^{2N}\det\left(\theta \right)}}\;e^{-\frac{1}{2}{\left(x-\bar{x}\right)}^{T}\theta^{-1}\left(x-\bar{x}\right)}$$

The entropy is defined by:

(24) $$S\;\left(t\right)=-\int P\left(x,t\right)\log\left(P\left(x,t\right)\right)dx$$

As shown by Landi and colleagues [53] and Gilson and colleagues [54], the rate of change of entropy can be decomposed as:

(25) $$\dot{S}\;\left(t\right)=\overset{.}{\underset{P}{H}}\;\left(t\right)-\dot{\phi }\;\left(t\right)=\int \frac{F^{T}\left(x\right)D^{-1}F\left(x\right)}{P\left(x\right)}\;dx+\int D^{-1}Jx.\;F\left(x\right)dx$$

### 4.4 Where the Stationary Probability Current **F**(*x*) Is Given by

(26) $$F\;\left(x\right)=D\;P\left(x\right)\theta^{-1}x+JxP\left(x\right)$$

From these equations, following the lead of Landi and colleagues, we can express the information processing (as the rate of the change in entropy):

(27) $$\dot{S}\;\left(t\right)=tr\left(D\theta^{-1}+J\right)$$

while the entropy production can be expressed as:

(28) $$\overset{.}{\underset{P}{H}}=tr\left(D\theta^{-1}+J\right)+tr\left(J^{T}D^{-1}J\theta +J\right)$$

and the model‐derived energy cost (measured as entropy flow out of the system) as follows:

(29) $$\dot{\phi }\;\left(t\right)=tr\left(J^{T}D^{-1}J\theta +J\right)$$

where *tr* is the trace of the matrix.

#### 4.4.1 Metastability

The metastability framework captures the spatiotemporal dynamics given as the variability of states of phase configurations as a function of time [14, 55], i.e., how the synchronization between the different nodes fluctuates across time [56]. Thus, the metastability can be measured as the standard deviation of the Kuramoto order parameter across time, defined by the following Equation (30):

(30) $$R\left(t\right)=\left|\sum_{k=1}^{n}e^{i\varphi_{k}\left(t\right)}\right|/n\;$$

where φ_*k*_(*t*) is the instantaneous phase of each narrowband BOLD signal at node *k*. The Kuramoto order parameter measures the global level of synchronization of the *n* oscillating signals. Under complete independence, the *n* phases are uniformly distributed and thus *R* is nearly zero, whereas *R* = 1 if all phases are equal (full synchronization).

Typically for fMRI, this is combined with the preprocessing of time series band‐pass filtered within the narrowband 0.008–0.08 Hz and compute the instantaneous phase φ_*k*_(*t*) of each narrowband signal *k* using the Hilbert transform. The Hilbert transform yields the associated analytical signals. The analytic signal represents a narrowband signal, *s*(*t*), in the time domain as a rotating vector with an instantaneous phase, φ(*t*), and an instantaneous amplitude, *A*(*t*), i.e., s(t)=A(t)cos(φ(t)). The phase and the amplitude are given by the argument and the modulus, respectively, of the complex signal *z*(*t*), given by *z* (*t*) =  *s*(*t*) + *i*.H[*s*(*t*)], where *i* is the imaginary unit and H[*s*(*t*)] is the Hilbert transform of *s*(*t*).

(31) $$H\;\left[s\left(t\right)\right]=-\frac{1}{\pi }\lim_{\varepsilon \to 0}\int_{\varepsilon }^{\infty }\frac{s\left(t+\tau \right)-s\left(t-\tau \right)}{\tau }d\tau$$

Please note that here we define the global metastability as the variability across time of the global Kuramoto parameter, that is the global level of synchronization.

#### 4.4.2 Quantum‐Like Effects in Coupled Oscillators

For our proof‐in‐principle investigation of studying QL states in the brain, we first investigate the local level using a network of coupled oscillators with the topology of 𝑘‐regular graphs. We concentrate on the class of directed graphs with no loops and no multiple edges, consisting of *n* vertices and *m* edges, connecting pairs of vertices. This can be described by a connectivity *n* x *n* matrix ***C***, with the weighted *m* edges. The spectrum of eigenvalues of ***C*** characterizes the emergent states. Scholes has shown that 𝑘‐regular random graphs, which belong to a class of specific expander graphs, exhibit strong resilience to decoherence when they possess a spectrum where the largest eigenvalue (or multiple eigenvalues) is separated from the rest of the spectrum [21]. In 𝑘‐regular random graphs every vertex is connected to 𝑘 edges, i.e., the degree of every vertex is 𝑘. Let the eigenvalues of this 𝑘‐regular graph be $\lambda_{0}\geq \lambda_{1}\geq \text{…}\;\geq \lambda_{n-1}$. For a 𝑘‐regular graph, the largest eigenvalue is λ_0_ =  *k* with corresponding eigenvector $\left(1,1,1,\text{…}\right)/\sqrt{n}$. The reason why 𝑘‐regular random graphs are candidates for the emergence of QL states is grounded in the findings from Scholes, using the Alon–Boppana Theorem [57, 58]. Specifically, the spectral gap, λ_0_ − λ_1_, defined as the difference between the largest eigenvalue and the second largest eigenvalue, is well‐separated from those of the remaining ‘random’ states.

When pruning of the edges in a 𝑘‐regular graph with the probability, *p_local_*, the large spectral gap associated with a QL‐state remains for low values of *p_local_*. High levels of pruning, corresponding to sufficiently large values of *p_local_*, cause the spectral gap—and consequently the QL state—to disappear. This is illustrated in the right panel of Figure 2A for a resilient 𝑘‐regular graph with *n*  =  40, *m*  =  20 and *p_local_* =  0.2 with its connectivity matrix showing the emergence of a large spectral gap and QL state. In contrast, when constructing a 𝑘‐regular graph using *p_local_* =  0.8 (left panel), the spectral gap disappears. This demonstrates that the quantum‐like behaviour of the 𝑘‐regular graph can be regulated by varying the level of pruning. We treat these k‐regular graphs as abstract modelling devices that generate the required spectral structure, and not as descriptions of the literal wiring of local cortical microcircuits. We further stress that the parameter values used here, including the number of nodes, the pruning probabilities and the inter‐network coupling, were chosen to produce a clear contrast in the spectral gap between the QL and non‐QL regimes. They are therefore a proof of concept at a chosen operating point rather than values that are natural or biologically motivated, and we make no claim that they are optimal.

Importantly, and similar to genuine quantum states, QL states can be expressed as vectors in a Hilbert space, allowing interference effects to be described in an equivalent formalism. Scholes showed that QL states emerging from the underlying graphs satisfy the following axioms [21]: (A) graphs exhibit an emergent state distinguishable in the eigenvalue spectrum; (B) the graphs are robust and stable to disorder; (C) the QL state is a two‐state system. In a network of oscillators sustained by these underlying graphs, the two states correspond to synchronization or no synchronization. Like genuine quantum states, interactions between different QL networks of oscillators can produce interference and superposition effects.

Khrennikov and colleagues [6] have shown how classical systems can produce probabilistic outcomes similar to those found in quantum mechanical systems. Following this line of work, Scholes proposed that networks of coupled oscillators can produce QL states that are robust to decoherence and exhibit interference and superposition effects within a Hilbert space framework. In other words, networks of coupled oscillators provide a natural implementation of QL states.

Combining two QL networks of coupled oscillators produces a QL bit [3]. Through cluster synchronization, such QL bits could generate two separate QL states that can interfere, with superposition governed by Hilbert space structure. Figure 2B (right panel) shows a QL bit consisting of two coupled *k*‐regular graphs (*n*  =  40, *m*  =  20 and *p_local_* =  0.2) whose vertices are interconnected with a probability of *p_inter_* =  0.1. As before, the local weights are 1, while the inter‐network weights are 0.17. The two QL states emerge due to the large underlying spectral gap, enabling the construction of a large classical system with quantum‐like superposition implemented through cluster synchronisation. In contrast, when manipulating the *k*‐regular graphs by increasing the pruning probability *p_local_* =  0.8,  these systems no longer exhibit QL states (shown in Figure 2B, left panel).

#### 4.4.3 Investigating QL Using Whole‐Brain Models of Coupled Oscillators

Given that *k*‐regular networks with coupled oscillators exhibit interference and superposition, we hypothesise that implementing these networks in whole‐brain models of empirical human brain activity will reveal quantum‐like computational properties. The dynamics of each brain region are governed by a *k*‐regular network of coupled oscillators, connected via anatomical structural connectivity. As a result, QL bits are distributed across paired brain regions, forming a distributed, connected, and overlapping architecture across the whole brain (see middle panels of Figure 2C for non‐QL and QL cases). Importantly, we use coupled Stuart‐Landau (SL) oscillators (i.e., as the normal form of a supercritical Hopf bifurcation), as these have been shown to provide excellent fits to whole‐brain activity [14, 18, 19]. At the level of the mathematics, the normal form of the supercritical Hopf bifurcation is the same as that of an exact dynamic mean field model of quadratic integrate‐and‐fire neurons [59, 60]. Crucially, the optimal working point of SL oscillators lies at the edge of the bifurcation, which allows linearization of the model [22]. This enables, for the first time, the construction of large‐scale analytical models of networks of networks capable of describing empirical human neuroimaging data.

Briefly, for each local brain region, we use a network of coupled oscillators connected by *k*‐regular network with *n*  =  40 and *m*  =  20, with two different levels of pruning, where QL effects emerge for low levels of pruning (*p_local_* =  0.2), and disappear for high levels of pruning (*p_local_* =  0.8). All brain regions are coupled using the anatomical structural connectivity matrix *C*, representing fibre density derived from diffusion MRI and normalized to 1. The SL oscillators between paired brain regions (*i*, *j*) are connected with the probability of *p_inter_* =  0.1 and scaled by *C_ij_*. Consequently, this builds a large‐scale network of networks ($\hat{C}$), consisting of the full brain network of *N* regions and where each region contains a *k*‐regular network with *n* regions, resulting in *M*  =  N*n. Figure 2C shows two examples of these large matrices $\hat{C}$ (with dimensions of *M* × *M*), corresponding to high pruning (non‐QL, left) and low pruning (QL, right). Figure 3 schematically illustrates the elements of the whole‐brain model.

#### 4.4.4 Quantum‐Like Effects in Empirical Brain Dynamics

Using the two different whole‐brain models applied to empirical neuroimaging data allowed us to directly assess quantum‐like effects. Figure 4 shows the fitting of whole‐brain models with non‐QL and QL local dynamics (i.e. with and without distributed spectral gaps as illustrated in Figure 2C, compare the spectral gaps shown in the right panels for non‐QL and QL conditions). As shown, there is a significant difference in the level of fit to large‐scale human resting‐state neuroimaging data from 1003 healthy participants, both in terms of correlation (Wilcoxon test indicated that the scores for QL (Median = 0.39, IQR = [0.39, 0.40]) were significantly different from Non‐QL (Median = 0.38, IQR = [0.37, 0.38]), Z = ‐9.25, *p* < 0.001, Cliff's delta = −0.76, Figure 4A) and quadratic error (Wilcoxon test indicated that the scores for QL (Median = 0.02, IQR = [0.02, 0.02]) were significantly different from Non‐QL (Median = 0.05, IQR = [0.05, 0.05]), Z = 12.22, *p* < 0.001, Cliff's delta = 1.00, *p* < 0.001, Figure 4B) between the simulated and empirical functional connectivity (FC) matrices. Figure 4C demonstrates that these differences in model fit are driven by significant differences in the underlying spectral gaps, as shown in the violin plots (Wilcoxon test indicated that the scores for QL (Median = 16.11, IQR = [16.00, 16.21]) were significantly different from Non‐QL $\left(\mathrm{Median}=5.17,\mathrm{IQR}=\left[5.14,5.21\right]\right),Z=-12.22,p$ < 0.001, Cliff's delta = −1.00). These values are calculated by summing spectral gap differences above a threshold (indicated by the red line) in the histograms of spectral gaps for non‐QL and QL models (see Figure 2C). Specifically, we calculated $\left(\lambda_{0}-\lambda_{1}\right)+\left(\lambda_{1}-\lambda_{2}\right)+\cdots$) over this threshold for both non‐QL and QL conditions.

Figure 4D shows renderings of regional spectral gaps projected onto the cortical surface for both non‐QL and QL models. Each region reflects the sum of pairwise spectral gaps between that region and all others. These maps reveal clear differences between the two regimes. Notably, in the QL case, there is substantial heterogeneity across brain regions, suggesting spatial variability in the strength of quantum‐like effects.

#### 4.4.5 Energy Cost of QL Computation

Figure 4E quantifies the model‐derived energy cost associated with whole‐brain modelling using the COCO framework [17], which integrates stochastic thermodynamics with whole‐brain modelling to analytically compute a model‐derived cost, entropy production, and information processing from neuroimaging data. This cost measures the thermodynamic price of information processing and communication in the Landauer sense rather than metabolic expenditure. The results show a substantial reduction in this model‐derived energy cost for QL models compared to non‐QL models. This suggests that quantum‐like dynamics may contribute to the efficiency with which the human brain processes and communicates information relative to artificial computational systems [17].

#### 4.4.6 The Role of Rare Long‐Range Connections in Brain Architecture

Previous research has identified a key feature of brain anatomy, namely rare long‐range connections that deviate from the exponential distance rule [7, 10]. Kennedy and colleagues demonstrated this rule using retrograde tract tracing in non‐human primates, showing that local connectivity can be largely explained by geodesic distance across the cortical surface [8, 9]. Given that human brain anatomy exhibits QL effects, we investigated whether removing these rare long‐range connections would significantly affect QL dynamics. To test this, we constructed individualized whole‐brain QL models for each of the 1003 participants, fitting empirical data using generative effective connectivity. We compared models with and without long‐range connections exceeding 80 mm. Using the spectral gap as a measure of QL effects, Figure 6A shows a significant reduction in spectral gaps when long‐range connections are removed (Wilcoxon test indicated that the scores with long‐range (Median = 0.72, IQR = [0.53, 0.92]) were significantly different from without long‐range (Median = 0.44, IQR = [0.35, 0.56], Z = 23.75, *p* < 0.001, Cliff's delta = 0.62). This demonstrates that long‐range connectivity amplifies quantum‐like dynamics in the model. We further examined the relationship between metastability (a measure of dynamical repertoire; see *Metastability* above) and QL effects (indexed by spectral gap). Figure 6B shows that the correlation between metastability and spectral gap is significantly stronger in models with long‐range connections (R = 0.42, *p* < 0.001) than in those without (R = 0.37, *p* < 0.001). These findings indicate that metastability, expressed as variability in cluster synchronization, is dynamically linked to QL spectral structure and is enhanced by long‐range anatomical connectivity.

#### 4.4.7 Turbulent Vortex Space: Spatiotemporal Metastability

Interference is most naturally read in the vortex space, the domain of spatiotemporal local synchronization, and turbulence provides the measure of it. Turbulence is not confined to fluids and appears in other physical systems as well, including coupled oscillators [61] and brains [13, 62, 63]. Yoshiki Kuramoto used coupled‐oscillator theory to account for turbulence in fluids [64], which suggests that turbulence may serve the efficient transfer of information as much as the transfer of energy. At the center of the construction is the Kuramoto local order parameter, a coupling‐weighted spatial average of the complex phase factor of the neighboring oscillators, which reports how tightly the local phases are synchronized around a chosen point. Amplitude turbulence follows as the standard deviation of the modulus of this quantity, and the definition applies to the empirical data of any physical system. Read in this way, the human brain displays a turbulent power law of the same form, consistent with a cascade of efficient information processing across scales [13, 62, 63, 65].

Turbulence is therefore obtained by forming the Kuramoto local order parameter and taking the standard deviation of its modulus over space and time [61]. The local Kuramoto order parameter *R* is thus computed by

(32) $$R\left(n,t\right)\;e^{i\nu_{n}\left(t\right)}=\sum_{p}\left[\frac{C_{np}}{\sum_{q}C_{np}}\right]e^{i\varphi_{p}\left(t\right)}\;$$

where φ_p_(t) are the phases of the spatiotemporal data, ν_n_(t) are the phases of the Kuramoto order parameter and *C_np_* is the local neighbourhood weighting exponential kernel between brain regions n and p given by

(33) $$C_{np}=e^{-\lambda \left(r\left(n,p\right)\right)}\;$$

where λ defines the spatial scaling and *r*(*n*, *p*) is the Euclidean distance between the brain regions n and p. We took λ = 0.18 mm^−1^, obtained by fitting the anatomical exponential rule to the dMRI tractography [62]. Turbulence, T, is then the standard deviation of the local Kuramoto order parameter over space and time:

(34) $$T\;=\langle R{\left(n,t\right)}^{2}\rangle -{\langle R\left(n,t\right)\rangle }^{2}$$

where the brackets 〈〉 denotes average across vertex space and time. Note, that the local Kuramoto order parameter *R*(*n*, *t*) in fact defines a new spatiotemporal dynamic in the so‐called vortex space.

An analytical equation is available for the evolution of the local Kuramoto order parameters, and it describes the dynamics of the vortex space on a partition of the original system, here the Schaefer1000 parcellation (*M*  =  1000) [65]. In this case, the local Kuramoto order parameter is defined in a partitioned Schaefer100 (*N* = 100) as follows:

(35) $$z_{\sigma }\left(t\right)r_{\sigma }\left(t\right)\;e^{i\vartheta_{\sigma }}=\frac{1}{\left|P\left(\sigma \right)\right|}\sum_{p\subset P\left(\sigma \right)}e^{i\hat{\varphi }\left(p,t\right)}$$

This uses a partition ℘ = (*P*(1).......*P*(*N*)) of the index set [1,…,M] by the Schaefer parcellation *N* = 100 of the original system defined in the Schaefer parcellation. Note that the *z*_σ_(*t*) expresses the vorticity in the partitioned space (“partitioned Local Kuramoto order parameters”) by averaging spatially the complex phase factor of the local phases spatially $\hat{\varphi }\left(p,t\right)$ from the BOLD time series signals in the fine Schaefer 1000 parcellation. The partitioned vorticity, *z*_σ_(*t*) reflects the level of synchronisation of the local phases in a given partition σ (here in the Schaefer100 parcellation) and is defining a partitioned version of the vortex space.

#### 4.4.8 Quantification of Classical Entanglement Through Interference

To establish that both structural and functional quantum‐like effects are present in brain dynamics, we introduced the Växjö interference measure. As before, we built individualized whole‐brain quantum‐like models for each of the 1003 participants and fitted them to the empirical data through generative effective connectivity. Here the local dynamics of each region are carried by single Stuart‐Landau oscillators rather than by k‐regular networks.

To perform probabilistic interference analysis, the partitioned vorticity *z*_σ_(*t*) was discretised, binning it in five states *S_i_* = [1, 2, 3, 4, 5]. This transformation allows for the definition of a discrete probability space where the state of a brain region σ in the Schaefer parcellation100 at any time *t* is categorised by its level of local collective oscillation.

In a classical stochastic system, the interaction between two regions *i* and 𝑗 should obey the Law of Total Probability (LTP). The LTP posits that the marginal probability of region *i* being in a specific state *m* can be perfectly reconstructed by summing the conditional probabilities of region *i* across all possible states *k* of region *j*, i.e.,

(36) $$P_{LTP}\;\left(\;S_{i}=m\right)=\sum_{k=1}^{5}P\left(S_{j}=k\right)\;P\left(\;S_{i}=m|S_{j}=k\right)$$

Any departure from this identity marks a contextual, quantum‐like interaction, in which the presence of region j shifts the probability distribution of region i in a non‐additive way. Within the Växjö framework for contextual probability, we defined the Växjö interference term δ_*ij*_ as the normalised violation of the law of total probability for the pair (*i*, *j*), starting from the absolute deviation, or interference residue, for each state *m*:

(37) $$\Delta_{m}=\;P\left(\;S_{j}=m\right)-\sum_{k=1}^{5}P\left(\;S_{j}=k\right)P\left(\;S_{i}=m|S_{j}=k\right)$$

The term was then weighted by the probabilistic importance of the interaction, normalizing the residue by the geometric mean of the joint probabilities over all states:

(38) $$\delta_{ij}=\sum_{k=1}^{5}\frac{\Delta_{m}}{\sqrt{\prod_{k=1}^{5}P\left(S_{j}=k\right)P\left(S_{i}=m|S_{j}=k\right)}}\;$$

The Växjö Interference Connectivity is the matrix of δ_*ij*_ values, and in the figures, it is summarised as the grand mean of δ_*ij*_ over all pairs, with pairs whose denominator approaches zero, and hence diverges, excluded.

## Author Contributions

**Yonatan Sanz Perl**: conceptualization, methodology, software, data curation, investigation, validation, formal analysis, supervision, resources, project administration, visualization, funding acquisition, Writing – original draft, Writing – review and editing. **Gustavo Deco**: conceptualization, methodology, software, data curation, supervision, formal analysis, validation, visualization, funding acquisition, investigation, writing – original draft, writing – review and editing, project administration, resources. **Shamil Chandaria**: conceptualization, methodology, writing – review and editing, writing – original draft. **Greg Scholes**: writing – review and editing, conceptualization, methodology, formal analysis. **Morten L. Kringelbach**: conceptualization, investigation, funding acquisition, writing – original draft, writing – review and editing, validation, methodology, visualization, project administration, formal analysis, software, data curation, supervision, resources. **Natasha Greenstein**: conceptualization, writing – review and editing.

## Conflicts of Interest

The authors declare no conflicts of interest.

## Data Availability

The data that support the findings of this study are openly available in the Human Connectome Project at https://db.humanconnectome.org, reference number 1U54MH091657.
